# An Enhanced Feature-Fusion Network for Small-Scale Pedestrian Detection on Edge Devices

**DOI:** 10.3390/s24227308

**Published:** 2024-11-15

**Authors:** Min Hu, Yaorong Zhang, Teng Jiao, Huijun Xue, Xue Wu, Jianguo Luo, Shipeng Han, Hao Lv

**Affiliations:** 1 Department of Medical Electronics, School of Biomedical Engineering, Air Force Medical University, Xi’an 710032, China; hummmin@163.com (M.H.); zhangyaorong181@xauat.edu.cn (Y.Z.); jiaoteng@fmmu.edu.cn (T.J.); xinyin20130419@163.com (H.X.); 2 School of Information and Control Engineering, Xi’an University of Architecture and Technology, Xi’an 710055, China; wuxue@xauat.edu.cn (X.W.); jianguo@xauat.edu.cn (J.L.)

**Keywords:** small-scale pedestrian detection, feature enhancement, gather-and-distribute mechanism, edge device

## Abstract

Small-scale pedestrian detection is one of the challenges in general object detection. Factors such as complex backgrounds, long distances, and low-light conditions make the image features of small-scale pedestrians less distinct, further increasing the difficulty of detection. To address these challenges, an Enhanced Feature-Fusion YOLO network (EFF-YOLO) for small-scale pedestrian detection is proposed. Specifically, this method employs a backbone based on the FasterNet block within YOLOv8n, which is designed to enhance the extraction of spatial features while reducing redundant operation. Furthermore, the gather-and-distribute (GD) mechanism is integrated into the neck of the network to realize the aggregation and distribution of global information and multi-level features. This not only strengthens the faint features of small-scale pedestrians but also effectively suppresses complex background information, thereby improving the accuracy of small-scale pedestrians. Experimental results indicate that EFF-YOLO achieves detection accuracies of 72.5%, 72.3%, and 91% on the three public datasets COCO-person, CityPersons, and LLVIP, respectively. Moreover, the proposed method reaches a detection speed of 50.7 fps for 1920 × 1080-pixel video streams on the edge device Jetson Orin NX, marking a 15.2% improvement over the baseline network. Thus, the proposed EFF-YOLO method not only boasts high detection accuracy but also demonstrates excellent real-time performance on edge devices.

## 1. Introduction

Pedestrian detection is a focal area of research in computer vision [1], with broad applications in intelligent surveillance [2], autonomous driving [3,4], smart robotics [5], and other fields. However, the characteristics of complex backgrounds, long distances, and low-light conditions make small-scale pedestrian detection relatively challenging, often leading to issues of missed or false detections. To better detect small-scale pedestrians against complex backgrounds, deep learning (DL)-based methods have gradually become the mainstream algorithms for pedestrian detection. However, because of the limitations of computational resources on edge devices, the deployment of current large DL models is difficult in practical applications. While some lightweight algorithm models can be deployed on edge devices, they often also suffer from problems such as delays and decreased detection frame rates. Therefore, achieving precise small-scale pedestrian detection in complex environments while accelerating model inference speed remains a significant challenge for the deployment of algorithm models on edge devices.

In recent years, numerous studies have been devoted to DL-based pedestrian-detection techniques, which can be primarily classified into two-stage pedestrian detection [6] and one-stage pedestrian detection [7]. Two-stage detection algorithms first generate region recommendations and then perform classification and regression [6], typically represented by R-CNN networks. As the first DL algorithm successfully applied to object detection, R-CNN [8] utilizes the powerful feature-learning capability of CNN to improve object-detection accuracy. However, with its slow inference speed, it is difficult to meet the demand of real-time detection. Aiming to improve the detection speed, He et al. [9] proposed SPPNet by adding spatial pyramid pooling between the final convolutional and the fully connected layer of R-CNN. SPPNet can accept input images of arbitrary size, which significantly improves detection speed. Subsequently, Girshick [10] proposed Fast R-CNN on the basis of R-CNN. The method further optimizes the feature-extraction process for each candidate region by introducing the ROI pooling operation. Subsequently, a multi-classification operation is performed using softmax, and finally the position of the bounding box is adjusted using a regression model to improve the detection speed and accuracy. Ren et al. [11] proposed Faster R-CNN, which is an important milestone for two-stage detection algorithms. Faster R-CNN introduces a region proposal network, which integrates candidate region generation, feature extraction, bounding-box regression, and classification into a single unified network. This enables end-to-end training and inference and significantly improves detection speed and overall performance. In addition, Mask R-CNN [12] extends Faster R-CNN by adding a mask branch for generating object masks. The ROI pooling operation is replaced with ROI align to address the alignment issue between the mask and the object in the original image. Oriented Mask R-CNN [13] enhances the efficiency and accuracy of detecting rotating or directionally oriented objects by simplifying the region proposal-generation process through the introduction of a midpoint offset representation, based on the Mask R-CNN. Although the two-stage detection algorithm excels in detection accuracy, it is still difficult to achieve real-time detection due to its large computational volume.

The one-stage algorithm simplifies the object-detection process by omitting the step of generating candidate regions and directly predicting the location and class of the object [7]. Typical representatives of this class of methods are mainly SSD [14,15,16,17,18] and YOLO [19,20,21,22,23,24,25,26]. SSD [14] extracts features on multiple feature layers of the pyramid structure and applies softmax and positional regression to determine the location and class of the object. However, SSD relies on lower-level feature information, leading to its poor performance in dealing with objects at different scales, especially for the detection of small objects. To solve this problem, Fu et al. [15] proposed DSSD, utilizing ResNet101 [27] as the base network and incorporating image feature fusion to improve the capability of extracting features. In addition, Jeong et al. [17] introduced R-SSD, which improves the recognition ability of small objects by enhancing the interaction of feature information between different convolutional layers. The feature-fusion SSD proposed by Cao et al. [19] adopts an integrated strategy to combine features from both high-level and low-level convolutional layers. This strategy not only improves the detection accuracy of small objects, but also enhances the overall perception of the network. In order to integrate features from different convolutional layers more efficiently, Li et al. [18] proposed FSSD. Different from the complex feature pyramid-construction method of the feature pyramid network (FPN) [28], FSSD adopts a more concise method to fuse the features of each layer after adjusting them to the same size by bilinear interpolation. This method improves the speed of fusion while achieving good detection results.

YOLO, as the first one-stage detection method, is one of the most successfully applied algorithms for pedestrian detection. Redmon et al. [19] first introduced YOLO in 2015, which is capable of predicting multiple bounding box (BBox) locations and categories simultaneously, marking the beginning of general-purpose object detection. YOLO can complete the object detection by a single forward propagation, which greatly simplifies the detection process and improves the detection speed. In 2017, Redmon [20] introduced YOLOv2, utilizing K-means clustering to derive more effective anchor templates from a training set. Nevertheless, YOLOv2 used features from the last convolutional layer, leading to the loss of a significant amount of information. Subsequently, YOLOv3 [21] was proposed with improvements on YOLOv2 by adopting the darknet-53 network architecture in place of the darknet-19 and employing a FPN for multi-scale detection. Although YOLOv3 adopted logistic regression instead of softmax, ensuring real-time performance while maintaining the accuracy of object detection, its performance was not effectively integrated with BBox. To further improve detection performance, YOLOv4 [22] and YOLOv5 [23] built upon YOLOv3 by integrating CSP and SPP structures, adaptive anchor calculations, and focus operations to enhance object-detection accuracy. YOLOv6 [24] and YOLOv7 [25] further improved detection performance by introducing the RepVGG [29] and efficient layer aggregation network (ELAN) modules, respectively. Inspired by the ELAN design of YOLOv7 [25], YOLOv8 [26] adopts a more gradient-rich C2f structure in place of the C3 structure and adjusts the channel numbers for models of varying scales, thus enhancing the detection performance of YOLOv8.

Despite significant progress in pedestrian detection by DL techniques in recent years, there are still obvious challenges in small-scale pedestrian detection. Existing methods perform poorly when dealing with complex backgrounds and low-light conditions, often resulting in missed or false detections. In addition, DL-based pedestrian-detection methods are usually accompanied by a large number of parameters and computations, leading to a relatively limited deployment of models for applications. Therefore, how to accurately detect the object without obvious visualization features and at the same time speed up the model inference is a major challenge at present. Through a review and analysis of commonly used object-detection algorithms, we propose an Enhanced Feature-Fusion YOLO network (EFF-YOLO) based on YOLOv8, aimed at improving the accuracy of small-scale pedestrian detection and deploying it on edge devices for real-time testing. The main contributions are as follows:

- A novel feature-enhanced fusion YOLO network is introduced. Utilizing a backbone based on the FasterNet block, this method enhances the extraction of spatial features from images while minimizing redundant operations. Meanwhile, the model size is optimized to facilitate deployment on edge devices for real-time detection.
- The gather-and-distribute (GD) mechanism is integrated into the neck to enhance faint features. By effectively aggregating and distributing information between global and multi-level features, this promotes efficient interaction of small-scale object features without introducing additional latency.
- Experiments demonstrate that EFF-YOLO outperforms baseline models in terms of detection accuracy on three public datasets, and achieves higher real-time detection frame rates on the edge device. This further validates the practical application potential of EFF-YOLO on edge devices.

The structure of this paper is organized as follows. Section 2 introduces the design of EFF-YOLO. In Section 3, the experiment details and results are presented to demonstrate the practicability of EFF-YOLO. Section 4 provides conclusions and outlines future research directions.

## 2. Methods

### 2.1. Overview of EFF-YOLO Architecture

**Figure 1:**
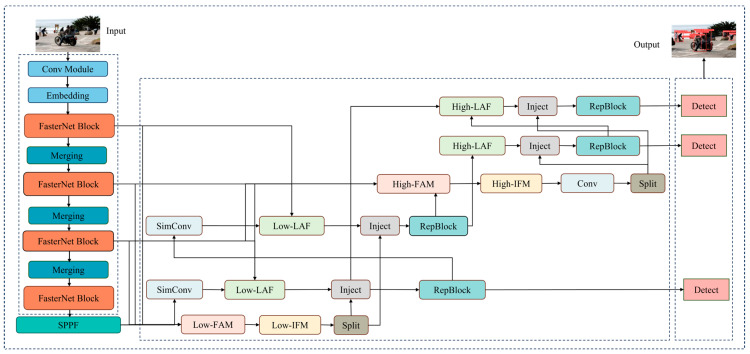
The architecture of EFF-YOLO.

Here, we propose EFF-YOLO based on YOLOv8 to improve the detection of small-scale pedestrians in complex scenes. In these scenarios, traditional models often struggle with accurately detecting and localizing small-scale pedestrians due to the high level of noise and the reduced resolution of distant objects. Our proposed method addresses these challenges through several key improvements. First, EFF-YOLO replaces the original C2f structure of backbone with FasterNet block [30] which has lower latency and higher throughput. The purpose is to reduce unnecessary computation and memory access while extracting efficient spatial features, resulting in higher operating speeds and lower latency on resource-limited devices. Then, we use the gather-and-distribute (GD) mechanism [31] instead of the original recursive method to capture finer-grained spatial information. This method can extract more important features in the detection process to enhance the perception of small-scale pedestrians. Finally, the anchor-free design in YOLOv8 is retained in the network head to ensure small object-detection capability. This design reduces the number of anchor frames by directly predicting the center and the ratio of width and height of the objects, thus further improving the detection accuracy and speed of the model. The model structure is shown in Figure 1.

### 2.2. Lightweight Backbone Network

The backbone is responsible for extracting features from the input and is the core of the pedestrian-detection network. YOLOv8 employs a parameter-sharing design in the backbone to improve the efficiency and generalization of the model. However, due to its parameters and complex structure, it still causes delays in the object-inference process. Given the limited performance of edge devices, this latency is particularly detrimental to the deployment and application of the model, especially in scenarios that require high real-time performance such as intelligent monitoring. Therefore, we have made the YOLOv8 algorithm lightweight with the ultimate goal of final deployment performance by deeply analyzing the hardware and software characteristics of the AI edge computing platform.

**Figure 2:**
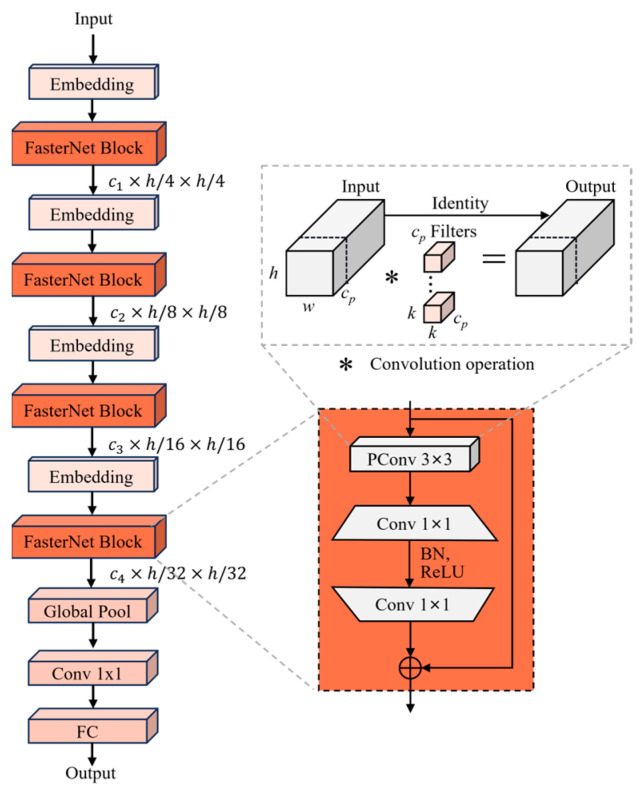
The structure of FasterNet.

In the network, we introduced a novel FasterNet [30] block to replace the YOLOv8 backbone. This block is based on partial convolution (PConv) and aims to solve the problems of redundant computation and frequent memory accesses. The structure is shown in Figure 2. The design of FasterNet consists of four main stages, each preceded by an embedding or merging layer for spatial down-sampling and extending the number of channels. Specifically, a 4 × 4 convolution with a stride of 4 is used for the embedding layer and a 2 × 2 convolution with a stride of 2 is used for the merging layer. In addition, the main components at each stage are stacked FasterNet blocks. The core component of each FasterNet block consists of a PConv layer for reducing redundant computations and memory accesses, and two 1 × 1 convolutional layers immediately following for further processing of features. These components collectively form an inverse residual block. Its middle layer expands the number of channels and adds skip connection to reuse input features. This design helps to retain important information in the deeper layers of the network and minimizes information loss.

The working principle of PConv, which is the core component, is shown in the right of Figure 2. PConv performs spatial feature extraction by applying a standard convolution on the input channel and leaving the remaining channels unchanged. For consecutive or regular memory accesses, the first or last continuous channel $c_{p}$ is computed as a representation of the entire feature. The FLOPs of PConv are calculated as follows:

(1) $$FLOPs_{PConv}=h\times w\times k^{2}\times c_{p}$$

where h and w represent the height and width, and k represents the convolutional kernel size. At the typical compression ratio $r=\frac{c_{p}}{c}=\frac{1}{4}$, PConv has only one-sixteenth of the FLOPs of a regular Conv. Moreover, PConv’s memory accesses are calculated as follows:

(2) $$MemoryAccess_{PConv}=h\times w\times 2c_{p}+k^{2}\times c_{p}^{2}\approx h\times w\times 2c_{p}$$

For $r=\frac{c_{p}}{c}=\frac{1}{4}$, PConv’s memory access is only one-fourth that of a regular convolution. In addition, batch normalization (BN) [32] is chosen for FasterNet because BN can be merged with the adjacent convolution layer to achieve faster inference while preserving the effect. GELU [33] is chosen for the activation layer.

### 2.3. Enhanced Feature-Fusion Module

**Figure 3:**
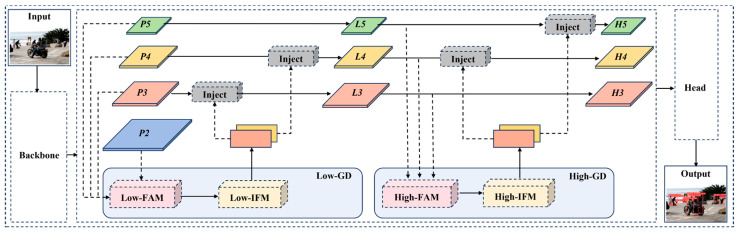
The neck network based on the GD mechanism.

The neck structure in YOLO is designed to integrate the multi-scale features extracted by the backbone. Traditional FPN and its variants are the most commonly used fusion methods. However, these methods have a significant drawback: when information needs to be fused across layers, the traditional FPN structure cannot ensure the complete transmission of information, which affects the overall effectiveness of information fusion to a certain extent. To address this issue, EFF-YOLO abandons the original recursive approach and incorporates an advanced GD mechanism [31] in the neck, as shown in Figure 3. This mechanism enhances feature fusion by collecting and integrating information from all levels and distributing it to different levels, thereby improving the information integration ability of the neck and avoiding the problem of information loss in traditional approaches.

The GD module consists of three key components: feature alignment module (FAM), information-fusion module (IFM), and information injection module (IIM). FAM is responsible for collecting and aligning features from different levels to ensure that feature maps at different levels are properly grouped together in subsequent processing. IFM further incorporates these features to produce global context information. This step helps capture multi-scale features. IIM distributes the global information generated by IFM to each level and injects it into the corresponding branches with simple attention operations to enhance detection capabilities. Meanwhile, low-stage GD (Low-GD) branch and high-stage GD (High-GD) branch are introduced to detect pedestrians at different scales. The improved structure of the neck is shown in Figure 3.

#### 2.3.1. Low-GD

**Figure 4:**
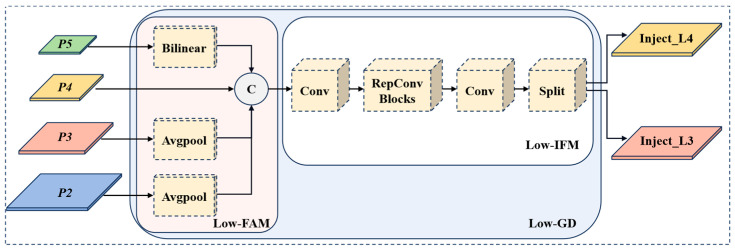
The structure of low-stage GD branch.

The output features P2, P3, P4, P5 from backbone are fused in the Low-GD module to obtain high-resolution spatial information that retain small-scale pedestrian features. As shown in Figure 4, average pooling (Avgpool) is used in low-stage FAM (Low-FAM) to adjust the size of all feature maps to the same as the feature P4, so as to promote information aggregation and reduce the amount of computation. We choose P4 as the object size for feature alignment while balancing speed and accuracy to ensure efficient processing of information. The features $F_{align}$ processed by Low-FAM are represented as follows:

(3) $$F_{align}=Low\text{\_}FAM\left(\left[P2,P3,P4,P5\right]\right)$$

The alignment feature $F_{align}$ then generates global information through low-stage IFM (Low-IFM). Low-IFM includes multi-layer reparameterized convolution blocks (RepBlock), convolution operation at input and output, and finally split operation. The global features are generated by using $F_{align}$ as an input through RepBlock and then they are divided into $F_{inj\text{\_}L3}$ and $F_{inj\text{\_}L4}$ in the channel dimension for fusion with features at different levels. The expression is as follows:

(4) $$F_{inj\text{\_}L3},F_{inj\text{\_}L4}=Split\left(RepBlock\left(F_{align}\right)\right)$$

**Figure 5:**
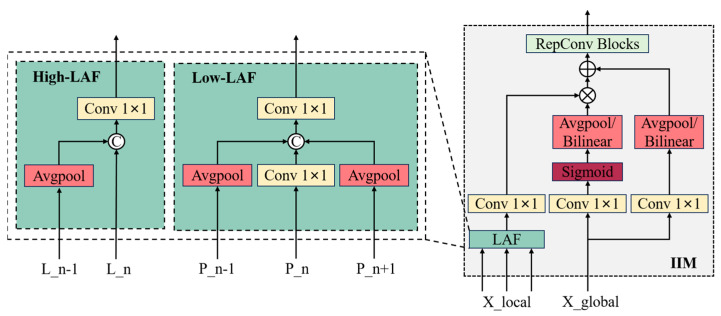
The details of IIM with LAF.

The global information is gathered through FAM and IFM and then the obtained global information is injected into different levels through IIM, as illustrated in Figure 5. IIM combined with lightweight adjacent layer fusion (LAF) increases the number of information flow paths between different levels by simplifying operations, thus improving performance without significantly increasing latency. The LAF module contains Low-LAF and High-LAF, which are used for low-level injection and high-level injection, respectively. Specifically, local information $F_{local}$ input into the module is processed by LAF and 1 × 1 convolutional layer. The global injection information $F_{inj}$ is processed by two different convolutional layers to obtain $F_{global\text{\_}embed}$ and $F_{global\text{\_}act}$. The expression for the operation is as follows:

(5) $$F_{global\text{\_}act\text{\_}Li}=Resize\left(Sigmoid\left(Conv_{act}\left(F_{inj\text{\_}Li}\right)\right)\right)$$

(6) $$F_{global\text{\_}embed\text{\_}Li}=Resize\left(Conv_{global\text{\_}embed\text{\_}Li}\left(F_{inj\text{\_}Li}\right)\right)$$

Subsequently, the global information and local information obtained by the attention mechanism to generate the fused feature map $F_{att\text{\_}fuse\text{\_}Li}$. Ultimately, $F_{att\text{\_}fuse\text{\_}Li}$ is further processed by RepBlock to generate the final feature $L_{i}$ for the subsequent task. The expression is as follows:

(7) $$F_{att\text{\_}fuse\text{\_}Li}=Conv_{local\text{\_}embed\text{\_}Li}\left(Bi\right)*F_{global\text{\_}act\text{\_}Li}+F_{global\text{\_}embed\text{\_}Li}$$

(8) $$L_{i}=RepBlock(F_{att\text{\_}fuse\text{\_}Li})$$

#### 2.3.2. High-GD

**Figure 6:**
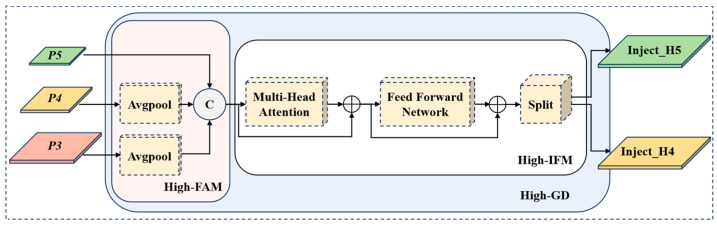
The structure of high-stage GD branch.

The features $\left\{L_{3},L_{4},L_{5}\right\}$ generated by Low-GD are processed in High-GD, as shown in Figure 6. In keeping with Low-GD, High-FAM is first used to align the size of features $\left\{L_{3},L_{4},L_{5}\right\}$ with the smallest size $L_{5}$ using Avgpool. The expression is as follows:

(9) $$F_{align}=High\text{\_}FAM\left(\left[L3,L4,L5\right]\right)$$

Then, $F_{align}$ is fused and decomposed by High-IFM. Unlike Low-IFM, High-IFM uses transformers to capture more complex dephasing and to understand contextual information in the image. In addition, to avoid the computational burden of the transformer, High-IFM uses Batch Normalization instead of Layer Normalization and ReLU instead of GELU to reduce the inference time. The workflow for High-IFM consists of three steps. First, fusion features are generated through the transformer block. Secondly, the number of channels for the features is reduced by 1 × 1 convolution. Finally, the fusion features are decomposed into $F_{inj\text{\_}H4}$ and $F_{inj\text{\_}H5}$ by a split operation. The expression is as follows:

(10) $$F_{inj\text{\_}H4},F_{inj\text{\_}H5}=Split\left(Conv1\times 1\left(Transformer\left(F_{align}\right)\right)\right)$$

Lastly, the obtained local features and global information are fused by IIM which is completely consistent with Low-GD. Therefore, the process of information injection can be expressed by the formula:

(11) $$F_{global\text{\_}act\text{\_}Hi}=Resize\left(Sigmoid\left(Conv_{act}\left(F_{inj\text{\_}Hi}\right)\right)\right)$$

(12) $$F_{global\text{\_}embed\text{\_}Hi}=Resize\left(Conv_{global\text{\_}embed\text{\_}Hi}\left(F_{inj\text{\_}Hi}\right)\right)$$

(13) $$F_{att\text{\_}fuse\text{\_}Hi}=Conv_{local\text{\_}embed\text{\_}Hi}\left(Li\right)*F_{global\text{\_}act\text{\_}Hi}+F_{global\text{\_}embed\text{\_}Hi}$$

(14) $$H_{i}=RepBlock(F_{att\text{\_}fuse\text{\_}Hi})$$

## 3. Experiments and Discussion

### 3.1. Datasets

In order to evaluate the ability of EFF-YOLO in small-scale pedestrian detection under a complex background, three representative datasets are selected in this experiment. The following are the details of the three datasets and Table 1 shows the specific division of the datasets.

COCO-person [34]: A sub-dataset consisting of images containing small-scale pedestrians is selected from the COCO dataset. This dataset includes small objects such as shoppers and vendors on the plaza and pedestrians on the pedestrian street. The object scenarios are rich and diverse and are suitable for small object pedestrian-detection tasks.

Citypersons [35]: The optimized diverse pedestrian dataset includes small-scale pedestrians from various cities, encompassing a wide range of contextual environments such as streets and squares. This diverse design enhances the model’s generalization capability, enabling it to perform more effectively across different environments.

LLVIP [36]: A dataset designed for pedestrian detection in low-light conditions. This dataset includes images of pedestrians at various street locations between 6 and 10 PM. Detecting pedestrians under these conditions poses greater challenges to the model, as low-light environments typically introduce increased noise and reduced contrast.

### 3.2. Experiment Settings

The training environment for this experimental model consists of Ubuntu 20.04 operating system with an RTX 3090 GPU. The deep learning framework used is Pytorch 1.13.1 and CUDA 11.7. For training, the batch size of input images is set to 16, and the input size is 640 × 480. To expedite the convergence speed, the initial learning rate is set to 0.01, the weight decay coefficient to 0.0005, and the momentum factor to 0.937, with the SGD employed for training. All models are trained for 120 epochs. Conversely, during testing, the original image size of the dataset is used as input to examine the model’s adaptability to different pixel inputs. The edge device used in the experiments is the NVIDIA Jetson Orin NX, featuring a 6-core Carmel ARM CPU, 1024-core NVIDIA Ampere CUDA cores, and 8 GB of RAM.

### 3.3. Evaluation Metrics

Commonly used evaluation metrics in pedestrian detection include mean average precision (mAP), precision (P), recall (R), model size, giga floating-point operations per second (GFLOPs), and frames per second (FPS).

mAP is a comprehensive metric that reflects both the precision and recall performance of a detection algorithm. It is calculated by averaging the precision values at different recall levels across multiple classes. The definition is as follows:

(15) $$mAP=\frac{1}{N}\sum_{I=1}^{N}\int_{0}^{1}P(R)d(R)$$

Precision measures the proportion of true positive detections among all positive predictions. It is defined as:

(16) $$P=\frac{TP}{TP+FP}$$

Recall measures the proportion of true positive detections among all actual positive instances. It is defined as:

(17) $$R=\frac{TP}{TP+FN}$$

where TP, FN, and FP indicate true positive, false negative, and false positive, respectively. N indicates the total number of categories.

Model size refers to the storage space required to store the model, typically measured in megabytes (MB). A smaller model size is beneficial for deployment in resource-constrained environments.

GFLOPs measure the computational complexity of the model, representing the number of billions of floating-point operations required per second. Lower GFLOPs indicate a more computationally efficient model.

FPS is a critical indicator of processing speed, representing the number of frames the model can process in one second. Higher FPS values indicate faster real-time performance.

These metrics collectively provide a comprehensive evaluation of the performance, efficiency, and practicality of pedestrian-detection algorithms.

### 3.4. Results

#### 3.4.1. Ablation Study

A series of ablation experiments are conducted to evaluate the effectiveness of each improvement module in EFF-YOLO. Specifically, by employing a control-variable method, we modify different components of the model across three datasets, enabling a systematic analysis of performance variations under various scenarios. Using YOLOv8n as the baseline, we progressively introduce the FasterNet block (FB) and the gather-and-distribute (GD) mechanism. The final model that integrates all these enhancements is referred to as EFF-YOLO. This approach allows us to assess the specific impact of individual modules on overall model performance while maintaining consistency in other conditions.

Effect of FB: By adopting a lightweight design that reduces redundant computations and memory accesses, the PConv-based FB enhances computational efficiency and makes the model more suitable for environments with limited computational resources. As shown in Table 2, the model size is reduced by 40.3% and the computational load is decreased by 39% after employing FB as the backbone. However, this reduction in model size and computational requirements comes at the cost of a slight decrease in accuracy.

Effect of GD: Subsequently, the GD mechanism is added to YOLOv8n. This mechanism aims to enhance the model’s detection capabilities through more efficient feature aggregation and information flow. The results, shown in Table 2, indicate that the introduction of this mechanism achieves mAP_0.5_ increases of 1.2% (to 71.8%), 1.1% (to 72.1%), and 0.3% (to 90.7%) on the three public datasets, respectively. It is important to note that the introduction of GD brings no increase in model size compared to the baseline.

Effect of Combining FB with GD: Ultimately, we integrate the GD mechanism with FB technology to develop EFF-YOLO. This model seeks to combine the strengths of both approaches, achieving a balance between peak performance and computational efficiency. Experimental results show that EFF-YOLO achieves 72.5% (1.9%↑ and 1.5%↑) mAP_0.5_ on both the COCO-person and Citypersons datasets. Meanwhile, EFF-YOLO maintains a more reasonable computational complexity and model size.

**Figure 7:**
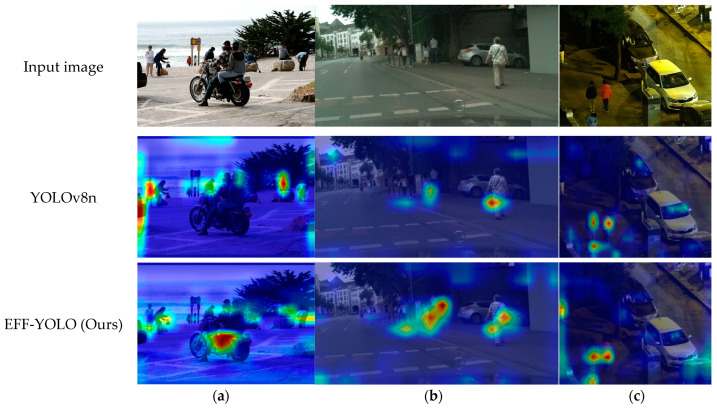
Comparison of visualized heatmaps from the EFF-YOLO and baseline on three datasets. (**a**) COCO-person; (**b**) Citypersons; (**c**) LLVIP.

To illustrate the difference in region of interest between EFF-YOLO and the baseline, we visualize the gradient heatmap in Figure 7. The small-scale pedestrians in the original image, combined with the lack of distinct pedestrian features in low-light conditions, make it challenging to discern edge features. However, by comparing the heatmaps, we observe that EFF-YOLO becomes more sensitive to the otherwise difficult-to-recognize pedestrian features after fusion with low-level features. Additionally, EFF-YOLO effectively suppresses interference from extraneous backgrounds, thereby achieving accurate localization of pedestrian features in images. This demonstrates that the improved algorithm not only enhances the recognition of subtle features but also improves detection performance in complex environments, thoroughly validating the effectiveness and superiority of the proposed method. Despite this, we observed that in dense small-scale pedestrian scenes on the COCO-person dataset, EFF-YOLO is less susceptible to interference from background information at the image edges and can more accurately localize human-related regions compared to YOLOv8n. However, our method still exhibits localization errors when dealing with heavily occluded pedestrians. This indicates that while EFF-YOLO excels at reducing background interference and enhancing feature recognition, further optimization is needed to improve detection accuracy and robustness in high-density and severely occluded scenarios.

#### 3.4.2. Comparison of Detection Accuracy Metrics

In order to validate the reliability and usefulness of EFF-YOLO for small-scale pedestrian detection, we conducted comparative experiments using five lightweight variants of the YOLO family, including YOLOv5n [23], YOLOv5s [23], YOLOv7-tiny [25], YOLOv8s [26], and YOLOv8n [26]. This evaluation aims to highlight the advantages of EFF-YOLO over these lightweight models in resource-constrained environments and real-time applications. Here, n denotes the smallest model variant nano, tiny denotes the smaller model variant, and s denotes the relatively larger but still lightweight model variant small. The experimental results are shown in Table 3. EFF-YOLO achieved 72.5% (2%↑), 72.3% (1.3%↑), and 91.0% (0.6%↑) mAP_0.5_ on the three benchmark datasets, respectively. Specifically, on the COCO-person dataset, YOLOv7-tiny achieved the highest mAP_0.5_, YOLOv8s achieved the highest mAP_0.5:0.95_ and precision, and EFF-YOLO achieved the highest recall value. On the Citypersons dataset, YOLOv8s reaches the highest mAP_0.5_ and mAP_0.5:0.95_, while EFF-YOLO achieves the highest precision. Furthermore, on the pedestrian-detection dataset LLVIP in low light, the mAP_0.5_, mAP_0.5:0.95_, and precision of EFF-YOLO are only 0.2%, 0.2%, and 1.4% lower than the highest metrics, which demonstrates a near-optimal level of performance in low-light conditions.

**Figure 8:**
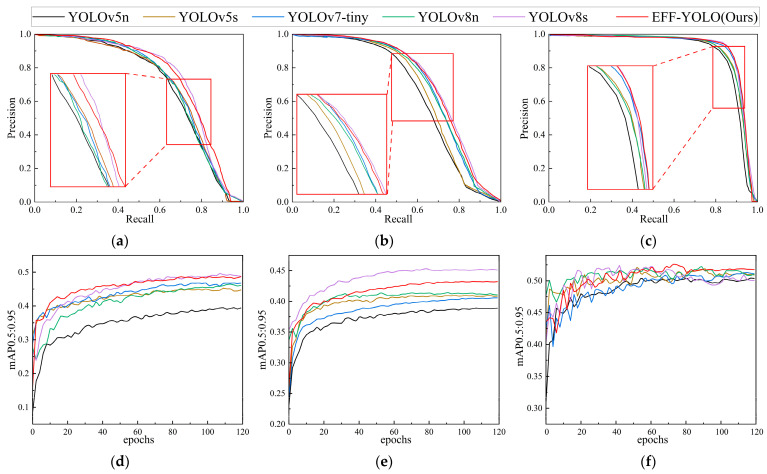
PR curves and mAP_0.5:0.95_ iteration curves of different models. (**a**) PR curve on COCO-person; (**b**) PR curve on Citypersons; (**c**) PR curve on LLVIP; (**d**) iterative curve of mAP_0.5:0.95_ on COCO-person; (**e**) iterative curve of mAP_0.5:0.95_ on Citypersons; (**f**) iterative curve of mAP_0.5:0.95_ on LLVIP.

In summary, our model achieves a significant accuracy gain compared to the baseline, but shows a slight drop in comparison to larger models such as YOLOv7-tiny and YOLOv8s. This phenomenon can be attributed to the lightweight architecture and enhanced feature-fusion module design we employ, resulting in a slight decrease in model performance in the acceptable 0–3% range. Notably, the performance of the EFF-YOLO in low-light environments is similar to that of the optimal model, indicating that our approach is well-suited for small-scale pedestrian detection in dim conditions. Meanwhile, we show the PR curves and mAP_0.5:0.95_ iteration curves of six algorithms on all datasets in Figure 8. Local magnification of the PR curve intuitively shows that the EFF-YOLO model has the lowest missing rate compared with YOLOv5n, YOLOv5s, YOLOv7-tiny, and YOLOv8n. In addition, EFF-YOLO achieves a similar miss rate to YOLOv8s at a lower parameter count. Among the mAP_0.5:0.95_ iteration curves of the three datasets, the mAP_0.5:0.95_ value of EFF YOLO is also higher than that of other models except YOLOv8s, indicating that EFF YOLO has superior performance in detecting small-scale pedestrians.

**Figure 9:**
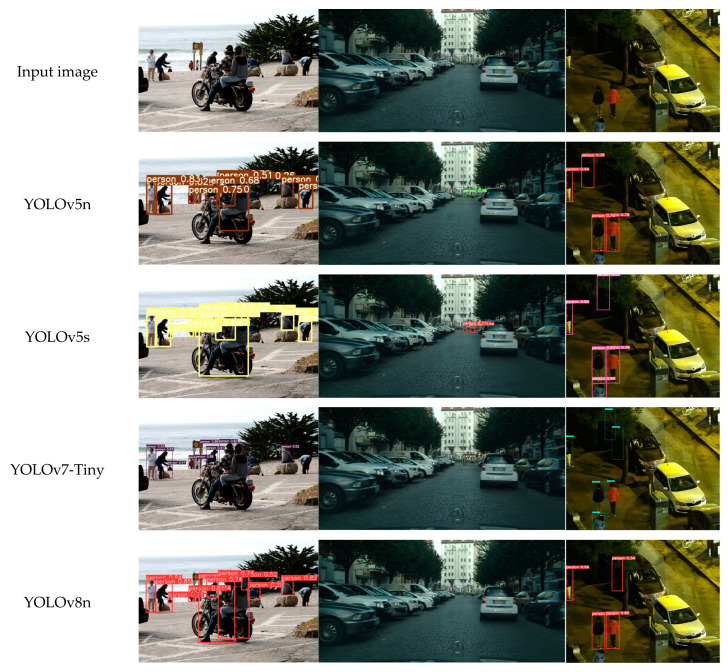

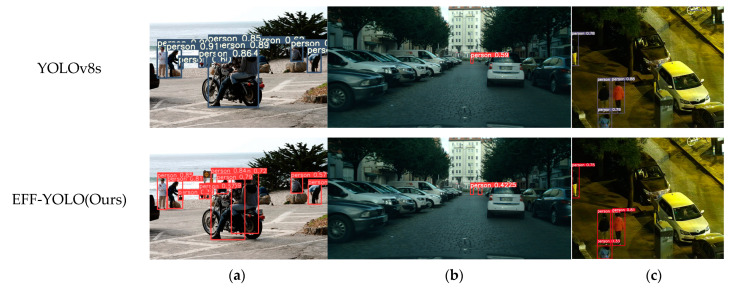
Visualization results of different models on three datasets. (**a**) COCO-person; (**b**) Citypersons; (**c**) LLVIP.

Through visual detection effects, we can intuitively evaluate the performance of different models in practical application scenarios, which helps to reveal the real performance of models in complex environments. The visualization of the model detection results is shown in Figure 9. Other algorithms generally suffer from missed detection or false detection when facing pedestrian images with different object scales. Specifically, the detection effect of the COCO-person dataset is shown in Figure 9a. EFF-YOLO can effectively identify and accurately locate objects for both small-scale pedestrians located far away and multiple human bodies on motorcycles. The visualization results of the Citypersons dataset are shown in Figure 9b. For small-scale pedestrians at the end of the street, only YOLOv5s and EFF-YOLO can accurately detect the pedestrians in the figure, while other algorithms cannot. Visualization results of the LLVIP dataset are shown in Figure 9c. When pedestrian-like features exist in images with weak light at night, only YOLOv8s and EFF-YOLO effectively suppressed interference information and accurately detected pedestrians. In summary, the EFF-YOLO model shows better results in pedestrian detection in low-light environment and small-scale pedestrian detection, and effectively completes the recognition task of this study.

#### 3.4.3. Comparison of Detection Inference Speed

In practical applications, it is very important to achieve a balance between the accuracy and speed of the model. This balance not only determines the usability of the model in the real world, but also directly affects the user experience and the adoption of the technology. The comparison of detection speeds of algorithms on all datasets is shown in Table 4. Specifically, on the COCO-person dataset, we obtained a mAP_0.5_ gain of 2% compared to YOLOv8n with an inference speed increase of only 0.3 ms. Meanwhile, compared with the highest accuracy YOLOv7-tiny, the inference time of EFF-YOLO is decreased by 25.2% and the detection rate is increased by 25.7 fps. On the Citypersons dataset, EFF-YOLO is superior in accuracy to other models except YOLOv8s and has a clear advantage in inference speed. On the LLVIP dataset, the accuracy and inference time of EFF-YOLO show clear advantages in all indicators. For example, EFF-YOLO shows only a 0.2% decrease compared to the highest-accuracy YOLOv5s and YOLOv7-tiny, but the inference time is accelerated by 8.9% and 37.4%, and the detection speed is improved by 9.7 fps and 40.7 fps, respectively. Our accuracy and speed improved by 0.3% and 4.5 fps, respectively compared to the lightest YOLOv5n. The experimental results show that EFF-YOLO maintains its light weight while still providing strong detection capabilities and superior performance in specific scenarios.

In addition, a comparative analysis of the memory required to load the model and the number of FLOPs required during the computation is shown in Table 5 to validate the advantages of the model for applications on resource-constrained devices. In this evaluation, we use the same input size of 640 × 480 to calculate and compare the model size and GFLOPs of each model. As can be seen from the data, EFF-YOLO performs particularly well in terms of model size, which is only 5.9 MB. Compared to other lightweight models there is a significant reduction; in particular, compared to YOLOv8s, the model size is reduced by 73.8%. Meanwhile, the score of EFF-YOLO on mAP_0.5_ reaches 72.5%, which is almost the same as YOLOv8s. This shows that EFF-YOLO achieves model miniaturization while maintaining high detection accuracy.

**Figure 10:**
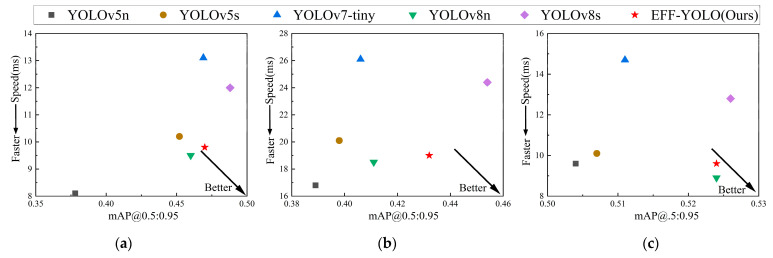
Comparison of algorithm speed and accuracy on three datasets. (**a**) COCO-person; (**b**) Citypersons; (**c**) LLVIP.

Figure 10 provides a comparative analysis of the speed–accuracy trade-off for six models across three datasets. EFF-YOLO demonstrates high accuracy and fast inference speed, indicating that the algorithm achieves faster detection while maintaining detection accuracy. This advantage makes EFF-YOLO more valuable in practical applications, especially for scenarios that require real-time processing of large amounts of data such as intelligent transportation systems and surveillance systems.

#### 3.4.4. Edge Deployment

**Figure 11:**
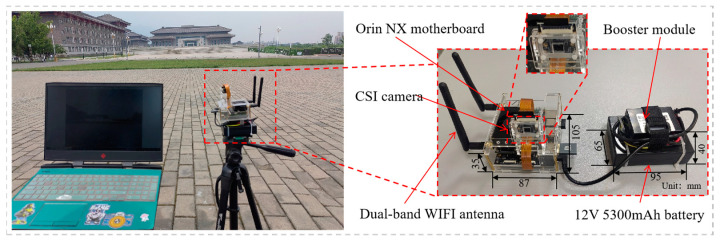
The experimental platform based on edge devices.

The optimized model is deployed on a Jetson Orin NX development board to test the real-time detection capability of the model. The deployment process uses TensorRT [37] to optimize the model in ONNX format and convert it to an engine file format specific to the NVIDIA platform. We also used DeepStream to build a video-analysis pipeline to simplify the complex video-processing logic. Finally, programs were written in C++ to call the converted models for real-time inference. To facilitate outdoor testing, NoMachine is used as a remote access solution to remotely control the Jetson development board over the network. The test platform and scenario are shown in Figure 11. The platform connects to a Sony IMX219 camera via a Camera Serial Interface (CSI) interface to obtain a live video stream as input data. All configurations run at 16-bit floating-point precision. The real-time detection scenarios include a school lawn and a main road, which mainly test the model’s detection ability in open areas and dynamic environments. The testing time covers both day and night to evaluate the robustness and accuracy of the model under different lighting conditions.

EFF-YOLO and the baseline model are deployed on the NVIDIA Jetson Orin NX for performance testing. To comprehensively evaluate the model performance, we test the detection frame rates for both image and video stream processing. For image detection, a series of standard image datasets are used to evaluate the model’s processing speed on static images. In the real-time camera detection tests, we evaluate the model’s real-time detection frame rates at both low resolution of 640 × 480 and high resolution of 1920 × 1080. The comparison results between EFF-YOLO and the baseline are shown in Table 6. The data indicate that the improved model outperforms the baseline in all aspects of performance. Specifically, EFF-YOLO has a smaller model size compared to YOLOv8n, providing an advantage on devices with limited storage space. When processing static images, the proposed method achieves a speed of 58.8 fps, representing an improvement of approximately 3.5% over the baseline model. In real-time video stream detection at a low resolution of 640 × 480, our model achieves 83 fps, which is a 12.2% increase in processing speed compared to the baseline. At high resolution of 1920 × 1080, the proposed method increases the frame rate by 6.7 fps compared to YOLOv8n, reaching 50.7 fps. Overall, EFF-YOLO provides notable improvements in both model size and detection speed, offering significant advantages in real-time detection applications. In particular, our method far exceeds the industry standard requirement of 30 fps in terms of frame rate, making it highly suitable for applications that demand high-performance real-time processing.

**Figure 12:**
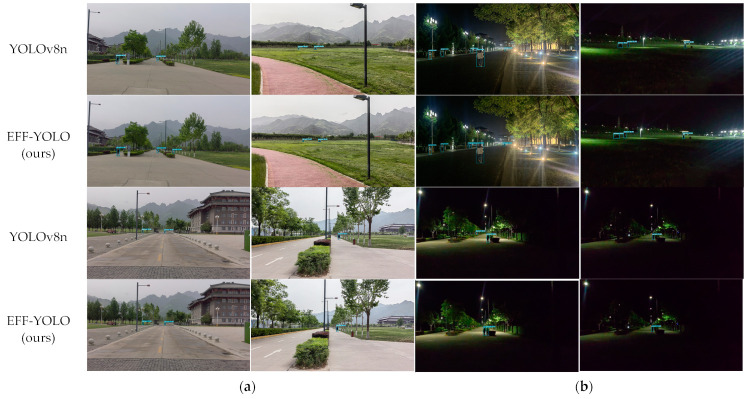
Visualization of small-scale pedestrian-detection results under different illumination on the Jetson Orin NX platform. (**a**) Day. (**b**) Night.

To validate EFF-YOLO in real-world application scenarios applied to the edge of equipment performance, we present the visualization results of pedestrian detection in various complex campus scenes in Figure 12. During the day, both EFF-YOLO and YOLOv8n demonstrate comparable detection performance in a variety of campus settings. Whether in open areas with clear visibility or in more cluttered environments with numerous obstacles, both models maintain high detection accuracy. However, EFF-YOLO shows a notable advantage in detecting small-scale pedestrians. For instance, in the first image, EFF-YOLO accurately detects a person riding a bicycle behind a flower bed, a scenario where YOLOv8n struggles. At night, the challenges increase due to reduced lighting and potential occlusions. Despite these conditions, EFF-YOLO continues to perform well, accurately detecting pedestrians even at a distance. This capability is particularly valuable for nighttime surveillance and safety applications, where traditional models might struggle due to poor lighting conditions. In contrast, the baseline YOLOv8n experiences more difficulties. On streets with significant exposure and tree interference, YOLOv8n produces a higher number of false negatives. Additionally, in the third row and third column image, YOLOv8n incorrectly identifies distant lights as pedestrians. These results further confirm that EFF-YOLO maintains excellent real-time detection performance while ensuring accuracy, making it a superior choice for applications such as campus security and crowd management, especially in challenging environments.

## 4. Conclusions

This paper addresses the challenge of small-scale pedestrian detection in complex backgrounds by proposing EFF-YOLO, a detection method that balances detection accuracy and model lightweight. Built upon YOLOv8n, EFF-YOLO incorporates advanced FasterNet and the GD mechanism to optimize the network. This design ensures that feature mappings not only contain semantic information from high-level features but also retain detailed information from low-level features, thereby enhancing the real-time and precise detection capabilities for small-scale pedestrians. The proposed algorithm was tested on the COCO-person, CityPersons, and LLVIP datasets. Experimental results show that, compared to YOLOv8n, EFF-YOLO improves detection accuracy by 2%, 1.3%, and 0.6%, respectively, and reduces the model size by 5%. On the edge device NVIDIA Orin NX, the model processes 1920 × 1080-pixel video frames from camera inputs at a smoothness of 50.7 fps, significantly exceeding the industrial standard of 30 fps. It is also able to accurately recognize pedestrians in dim lighting at night in the deployed applications. Moreover, when compared with other classic detection networks, EFF-YOLO exhibits superior results in terms of mAP, model parameters, and FPS. Overall, EFF-YOLO strikes a balance between accuracy and detection speed, effectively addressing the issues of small-scale pedestrian detection. We hope this research will contribute to advancing the application of edge intelligence terminals in areas such as intelligent surveillance, autonomous driving, and smart robotics.

## Figures and Tables

**Table 1:** Different subset divisions of the three datasets.

Dataset	Train	Validation	Test	Size
COCO-person	6192	774	774	640 × 480
Citypersons	2975	500	1575	2048 × 1024
LLVIP	12,025	2463	1000	1280 × 1024

**Table 2:** Ablation results of different modules.

Dataset	Yolov8n	FB	GD	mAP_0.5_	mAP_0.5:0.95_	P	R	Model Size	GFLOPs
COCO-person	√			0.706	0.460	0.746	0.635	6.2 M	8.2
	√	√		0.668	0.431	0.642	0.603	**3.7 M**	**5.0**
	√		√	0.718	0.468	0.777	0.662	6.2 M	17.6
	√	√	√	**0.725**	**0.470**	**0.778**	**0.672**	5.9 M	11.5
Citypersons	√			0.710	0.411	0.779	0.607	6.2 M	8.2
	√	√		0.625	0.364	0.709	0.520	**3.7 M**	**5.0**
	√		√	0.721	0.432	0.783	0.617	6.2 M	17.6
	√	√	√	**0.725**	**0.432**	**0.802**	**0.605**	5.9 M	11.5
LLVIP	√			0.904	0.524	0.919	0.825	6.2 M	8.2
	√	√		0.842	0.456	0.824	0.768	**3.7 M**	**5.0**
	√		√	0.907	0.526	0.908	0.836	6.2 M	17.6
	√	√	√	**0.902**	**0.524**	**0.923**	**0.826**	5.9 M	11.5

Bold represents the best results.

**Table 3:** Comparison of detection accuracy on three datasets.

Method	COCO-Person				Citypersons				LLVIP			
	mAP_0.5_	mAP_0.5:0.95_	P	R	mAP_0.5_	mAP_0.5:0.95_	P	R	mAP_0.5_	mAP_0.5:0.95_	P	R
YOLOv5n	0.550	0.379	0.526	0.401	0.679	0.389	0.768	0.581	0.907	0.504	0.905	0.849
YOLOv5s	0.668	0.452	0.768	0.637	0.674	0.398	0.782	0.582	**0.912**	0.507	0.909	0.862
YOLOv7-tiny	0.750	0.469	0.781	0.669	0.718	0.406	0.767	**0.633**	**0.912**	0.511	0.916	0.852
YOLOv8n	0.705	0.460	0.746	0.635	0.710	0.411	0.779	0.607	0.904	0.524	0.919	0.825
YOLOv8s	**0.726**	**0.488**	**0.804**	0.643	**0.734**	**0.454**	0.797	0.631	0.907	**0.526**	**0.937**	**0.867**
EFF-YOLO (ours)	0.725	0.470	0.778	**0.672**	0.723	0.432	**0.802**	0.605	0.910	0.524	0.923	0.826

Bold represents the best results.

**Table 4:** Comparison of inference speed on three datasets.

Method	COCO-Person @640 × 480			Citypersons @2048 × 1024			LLVIP @1280 × 1024		
	mAP0.5	FPS	Speed (ms)	mAP_0.5_	FPS	Speed (ms)	mAP_0.5_	FPS	Speed (ms)
YOLOv5n	0.550	**123.5**	**8.1**	0.679	**59.5**	**16.8**	0.907	**104.2**	**9.6**
YOLOv5s	0.668	98.0	10.2	0.674	49.8	20.1	**0.912**	99.0	10.1
YOLOv7-tiny	0.750	76.3	13.1	0.718	38.3	26.1	**0.912**	68.0	14.7
YOLOv8n	0.705	105.3	9.5	0.710	54.1	18.5	0.904	112.4	8.9
YOLOv8s	**0.726**	83.3	12.0	**0.734**	41.3	24.4	0.907	78.1	12.8
EFF-YOLO (ours)	0.725	102.0	9.8	0.723	52.6	19	0.910	108.7	9.2

Bold represents the best results.

**Table 5:** Comparison of different model complexity.

Method	mAP_0.5_	Model Size	GFLOPs
YOLOv5n	0.550	**3.7 M**	**5.2**
YOLOv5s	0.668	13.7 M	15.8
YOLOv7-tiny	**0.750**	12.3 M	13.2
YOLOv8n	0.705	6.2 M	8.2
YOLOv8s	0.726	22.5 M	28.2
EFF-YOLO (ours)	0.725	5.9 M	12.5

Bold represents the best results.

**Table 6:** FPS comparison between EFF-YOLO and baseline on the Jetson Orin NX platform.

Method	Model Size	Picture (FPS)	Real-Time Video (FP16, FPS)	
		640 × 480	640 × 480	1920 × 1080
YOLOv8n	6.2	56.8	74.0	44.0
EFF-YOLO (ours)	**5.9**	**58.8**	**83** **.0**	**50.7**

Bold represents the best results.

## Data Availability

The COCO-person dataset used in the experiments can be found at http://mscoco.org (accessed on 15 March 2024), The Citypersons dataset can be found at https://www.cityscapes-dataset.com (accessed on 15 March 2024), The LLVIP dataset can be found at https://bupt-ai-cz.github.io/LLVIP (accessed on 15 March 2024).
